# Web-Based Evaluation System to Measure Learning Effectiveness in Kampo Medicine

**DOI:** 10.1155/2016/2043535

**Published:** 2016-09-22

**Authors:** Norio Iizuka, Koichiro Usuku, Hajime Nakae, Makoto Segawa, Yue Wang, Kahori Ogashiwa, Yusuke Fujita, Hiroyuki Ogihara, Susumu Tazuma, Yoshihiko Hamamoto

**Affiliations:** ^1^Department of Kampo Medicine, Graduate School of Biomedical & Health Sciences, Hiroshima University, 1-2-3 Kasumi, Minami-ku, Hiroshima 734-8551, Japan; ^2^Department of Kampo Medicine, Yamaguchi University Hospital, 1-1-1 Minami-Kogushi, Ube, Yamaguchi 755-8505, Japan; ^3^Medical Information Science and Administrative Planning, Kumamoto University Hospital, 1-1-1 Honjo, Chuo-ku, Kumamoto City 860-8556, Japan; ^4^Department of Emergency and Critical Care Medicine, Akita University School of Medicine, 1-1-1 Hondo, Akita 010-8543, Japan; ^5^Media and Information Technology Center, Organization for Academic Information, Yamaguchi University, 1-1-1 Minami-Kogushi, Ube, Yamaguchi 755-8505, Japan; ^6^Liberal and General Education Center, Utsunomiya University, 350 Mine-machi, Utsunomiya, Tochigi 321-8505, Japan; ^7^Graduate School of Sciences and Technology for Innovation, Yamaguchi University, 2-16-1 Tokiwadai, Ube, Yamaguchi 755-8611, Japan; ^8^Graduate School of Medicine, Yamaguchi University, 2-16-1 Tokiwadai, Ube, Yamaguchi 755-8611, Japan; ^9^Department of General Internal Medicine, Hiroshima University Hospital, 1-2-3 Kasumi, Minami-ku, Hiroshima 734-8551, Japan

## Abstract

Measuring the learning effectiveness of Kampo Medicine (KM) education is challenging. The aim of this study was to develop a web-based test to measure the learning effectiveness of KM education among medical students (MSs). We used an open-source Moodle platform to test 30 multiple-choice questions classified into 8-type fields (eight basic concepts of KM) including “qi-blood-fluid” and “five-element” theories, on 117 fourth-year MSs. The mean (±standard deviation [SD]) score on the web-based test was 30.2 ± 11.9 (/100). The correct answer rate ranged from 17% to 36%. A pattern-based portfolio enabled these rates to be individualized in terms of KM proficiency. MSs with scores higher (*n* = 19) or lower (*n* = 14) than mean ± 1SD were defined as high or low achievers, respectively. Cluster analysis using the correct answer rates for the 8-type field questions revealed clear divisions between high and low achievers. Interestingly, each high achiever had a different proficiency pattern. In contrast, three major clusters were evident among low achievers, all of whom responded with a low percentage of or no correct answers. In addition, a combination of three questions accurately classified high and low achievers. These findings suggest that our web-based test allows individual quantitative assessment of the learning effectiveness of KM education among MSs.

## 1. Introduction

The Japanese have created their own unique medical system, known as Kampo Medicine (KM), based on traditional Chinese medicine (TCM), which was likely introduced to Japan directly or by way of Korea around the 5th or 6th century. In 1993, about 70% of the estimated 200,000 physicians practicing in Japan prescribed Kampo formulae in daily clinical practice [1], and this number has continued to increase, with a current prevalence of approximately 90% [2].

It is notable that 148 Kampo formulae are government-regulated prescription drugs listed in the Japanese national health insurance system and can therefore be prescribed by physicians in Japan. These formulae are fixed combinations of high quality herbs in proportions standardized based on the classical TCM literature [1–4]. In addition, the Japanese national health insurance system makes it possible to use Kampo formulae and various Western drugs concomitantly in daily clinical practice. This medical system is specific for Japan and is not observed in other Asian countries such as China and South Korea.

After the announcement in 2001 by the Japanese Ministry of Education, Culture, Sports, Science and Technology to expand the use of KM, it has been integrated into the medical education programs of all 80 of Japan's medical universities and colleges [5]. However, several problems remain to be solved in terms of KM education in Japan. Arai et al. [6] identified various differences between the 80 Japanese medical universities and colleges in both the number and length of class meetings for KM education, the presence or absence of full-time KM instructors, and the content of the curricula and textbooks used for KM education. Notably, in their survey report [6], 15 (19%) of the 80 institutions provided KM education based on TCM as opposed to KM theories. These heterogeneous education programs in Japan hinder the desire of medical students (MSs) who consider it necessary to have opportunities to continue learning KM after graduation, thereby delaying the diffusion of KM in Japan. The main problems faced by Japanese KM instructors are those in terms of the best content and purpose for the provision of KM education. Taken together, the development of a robust evaluation system is required to account for differences in KM education among Japan's medical universities and colleges. Therefore, the aim of this study was to develop a web-based test to measure the learning effectiveness of KM lectures among MSs.

## 2. Materials and Methods

In 2015, we offered seven KM classes to all 117 fourth-year MSs (74 men, 43 women; mean age ± standard deviation [SD], 23.6 ± 3.0 years) at Yamaguchi University School of Medicine. Each class was about 90 minutes. The content of the lectures consisted of the following 8-type fields (eight basic concepts of KM): “qi-blood-fluid” theory; diagnosis based on concepts of the eight guiding factors and the six stages of disease transformation; “five-element” theory; pattern-based diagnostic procedures (*four physical examinations of Kampo Medicine*); pattern-based therapeutic theory (*ho-sho sotai*); the pharmacology of Kampo formulae and their components; evidence-based KM; and the history of KM. After the seven classes, all MSs took our web-based test.

The following are contents of the lectures. Regarding “qi-blood-fluid” theory, we taught that the three elements served as a potent determinant for sho (Kampo patterns) in prescribing Kampo formula in the chronic condition. In particular, we highlighted the significance of qi (i.e., life energy), which is sourced from food and air and can move blood and fluid. For diagnosis in KM, several parameters are also used to determine sho, including yin-yang, deficiency-excess, cold-heat, and interior-exterior, which correspond to the eight guiding factors. This theory is considered significant to assess and better understand patient patterns in the whole body as well as “qi-blood-fluid” theory. We taught that the diagnosis was based on the six stages of disease transformation in the treatment of patients at an acute phase. In the “five-element” theory, it is considered that everything in nature consists of five elements with different property. These five elements correspond to five parenchymatous viscera, liver, heart, spleen, lung, and kidney, each of which is considered a functional unit. The above sho is determined by pattern-based diagnostic procedures (four physical examinations of Kampo Medicine) consisting of visual examination, examination by physician's sense, interview, and palpation examination. Pattern-based therapeutic theory (*ho-sho sotai*) means that the Kampo formulae suitable for patient can be determined by diagnosing sho reflecting the current condition. For the pharmacology of Kampo formulae and their components, we explained the pharmacology in the two viewpoints of Kampo and Western medicines. We taught only the pharmacology actions proven statistically in the educational program for evidence-based KM. For the history of KM, we taught that KM was based on traditional Chinese medicine (TCM) that was likely introduced to Japan directly or by way of Korea around the 5th or 6th century; during the Edo period (17th to 18th century), KM evolved uniquely as holistic medicine; in the Showa period (i.e., 1960s), KM gained the current popularity in support with integration into Japan's health care system.

We used a Moodle platform [7] to administer a test composed of 30 multiple-choice questions classified into the 8-type fields described above. For each question, MSs could choose one answer. We graded MSs on a scale of 100 (i.e., 3.3 points for each correct answer). There was some overlap for the 8-type fields among the 30 questions. We established a 30-minute time limit to complete the test. All tests were automatically graded by the Moodle-based system, which we also programed to compute the correct answer rates and create a portfolio regarding the 8-type fields for each MS. In the preliminary experiments (data not shown), we evaluated the true intelligibility of medical student by comparing the scores of test consisting of multiple-choice questions and those of a written examination. We found that easier multiple-choice questions could fail to evaluate the intelligibility precisely. Thus, it is necessary to weigh the relative difficulty of the questions. We therefore set up this web test so that the mean score becomes approximately 30/100.

MSs who scored higher than the mean + 1SD (*n* = 19) and those who scored lower than the mean − 1SD (*n* = 14) were defined as high and low achievers, respectively. To examine differences between high and low achievers in proficiency patterns for the 8-type fields, we performed cluster analysis by applying a nonlinear mapping technique developed by Sammon [8, 9] to the data. Briefly, MSs were located in the 8-dimensional (8D) space projected by the correct answer rates for questions regarding the 8-type fields. Next, the 8D space was converted into a 2D space using a nonlinear mapping tool, and the MS distributions were visualized. Interactive pattern analysis [10] makes it possible to find clusters identifiable by visual examination of the 2D configuration. To identify the questions that most strongly affected the learning effectiveness of MSs, we adopted the proficiency index (PI), which is the difference between the high and low achievers in the correct answer rate for each question. The PI is calculated by subtracting the correct answer rate for the low achievers from that in the high achievers.

Continuous variables between two groups were analyzed using Student's *t*-test, and those between three groups were analyzed using analysis of variance (ANOVA) followed by the Scheffe test. A *P* value < 0.05 was considered significant.

Since our current research involved neither patients' data nor intervention, we judged that this was exempt from the requirement for formal ethical approval of the Helsinki Declaration. However, it is important to inform participants (i.e., medical students) of the aims and contents of this study. We gave them a detailed explanation for this study and obtained the informed consent when they took our web-based test. On the basis of the Helsinki Declaration, it is our duty to protect confidentiality of personal information of the participants; therefore, the collected data for the test were processed and analyzed anonymously. All of these steps were performed securely by the research secretariat.

## 3. Results

The mean scores (±SD) on the web-based test were 30.2 ± 11.9 (range, 6.6–62.7). No significant differences were observed in mean scores between male and female MSs (29.3 ± 11.7 versus 31.7 ± 12.2, resp.; Student's *t* -test) (Table 1). MSs ≥24 years of age scored significantly higher than those <24 years of age (34.1 ± 13.6 versus 28.8 ± 11.0, resp.; *P* < 0.05; Student's *t-*test).

Among the 117 fourth-year MSs, 67 had passed typical university entrance examinations, 40 had been admitted based on recommendations, and 10 had been admitted to the undergraduate program. A significant association was found between test scores and the type of admission (*P* < 0.00001; ANOVA). MSs who had been admitted to an undergraduate program scored significantly higher (46.9 ± 9.2) than those who had passed typical university entrance examinations (28.8 ± 11.2; *P* < 0.00001; Scheffe test) and those who had been admitted based on recommendations (28.4 ± 10.4; *P* < 0.00001; Scheffe test) (Table 1).

**Figure 1:**
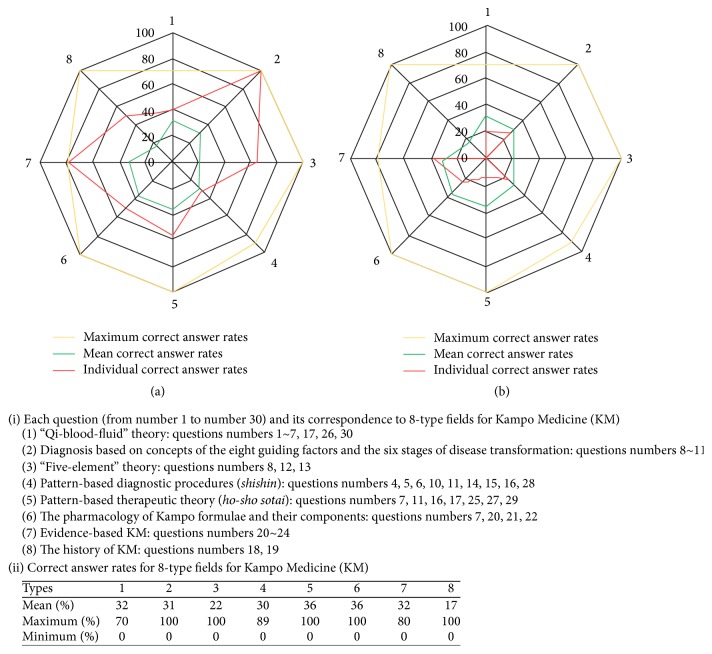
(a) High achiever with 53 points. (b) Low achiever with 20 points. Representative portfolios shown according to correct answer rates for questions on the following 8-type fields of Kampo Medicine (KM): “qi-blood-fluid” theory; diagnosis based on concepts of the eight guiding factors and the six stages of disease transformation; “five-element” theory; pattern-based diagnostic procedures (*four physical examinations of Kampo Medicine*); pattern-based therapeutic theory (*ho-sho sotai*); the pharmacology of Kampo formulae and their components; evidence-based KM; and the history of KM. Red lines in (a) and (b) show the correct answer rates (on a scale of 100) for a high achiever with 53 points and a low achiever with 20 points, respectively. Yellow and green lines show the maximum and minimum correct answer rates, respectively, among the 117 MSs included in this study. The high and low achievers appeared to have different strengths and weaknesses in terms of the 8-type fields of KM. Patterns of correct answer rates in the 8-type fields of Kampo Medicine (KM) in represntative medical students.

Correct answer rates for 8-type fields of “qi-blood-fluid” theory; diagnosis based on concepts of the eight guiding factors and the six stages of disease transformation; “five-element” theory; pattern-based diagnostic procedures (*four physical examinations of Kampo Medicine*); pattern-based therapeutic theory (*ho-sho sotai*); the pharmacology of Kampo formulae and their components; evidence-based KM; and the history of KM were 32%, 31%, 22%, 30%, 36%, 36%, 32%, and 17%, respectively (Figure 1). For the 8-type fields, 117 patterns of learning effectiveness specific to individual MSs were found, as shown in Figure 1. All test results were given to each MS directly in the form of a KM proficiency portfolio for individualized education.

**Figure 2:**
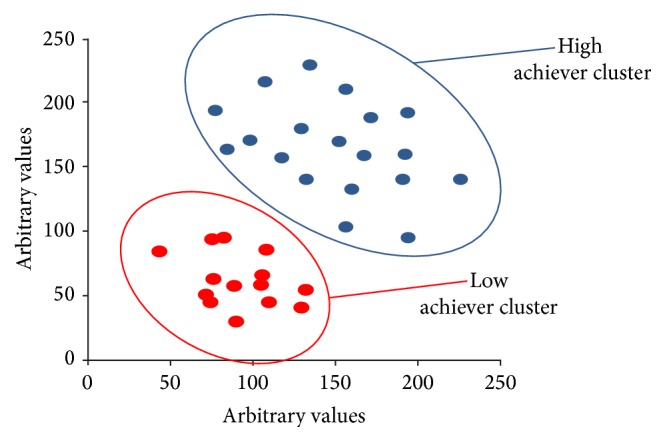
MSs who scored higher than the mean + 1SD (*n* = 19) and those who scored lower than the mean − 1SD (*n* = 14) were defined as high and low achievers, respectively. Values on the vertical and horizontal axes were computed arbitrarily using a nonlinear mapping tool (see [8] and [9]). Briefly, we have a set of medical students (MSs) in the 8-dimensional space corresponding to the correct answer rate of the 8-type fields of KM. Initially we choose at random a set of MSs in the 2-dimensional space. The set is the initial configuration of the 2-dimensional space. Next we compute all distances between the MSs in the 2-dimensional space. The next step in the algorithm is to adjust the vectors of MSs in the 2-dimensional space so that the configuration of MSs in the 2-dimensional space can well fit that of the MSs in the 8-dimensional space. This is achieved by carrying out a steepest descent procedure. As a result, we get the optimal configuration of MSs in the 2-dimensional space in this figure. In Sammon's algorithm, the configuration of MSs is essential. Hence, vertical axis and horizontal axis in the figure have no physical means. High (blue) and low (red) achievers classified according to cluster analysis using correct answer rates for 8-type fields of KM.

**Figure 3:**
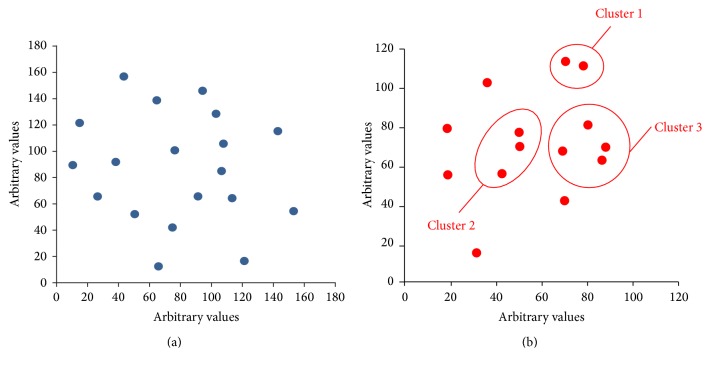
No new clusters were identified within the high achiever cluster based on proficiency patterns. In contrast, three new clusters were identified within the low achiever cluster; all of the students in the new clusters had a lower percentage of or no correct answers for questions on the history of KM. Intracluster distribution of high (a) and low (b) achievers according to cluster analysis using correct answer rates for 8-type fields of KM.

When defining MSs with points higher than mean + 1SD and lower than mean − 1SD as high and low achievers, respectively, clear divisions were seen between the high and low achievers based on cluster analysis using the correct answer rates (Figure 2). Next, we focused our investigation on each cluster of high and low achievers (Figures 3(a) and 3(b)). Although no new clusters were found within the high achiever cluster based on the proficiency pattern data, three new clusters were found within the low achiever cluster; all of the students in the new clusters had a lower percentage of or no correct answers for questions on the history of KM (Figure 3(b)).

**Figure 4:**
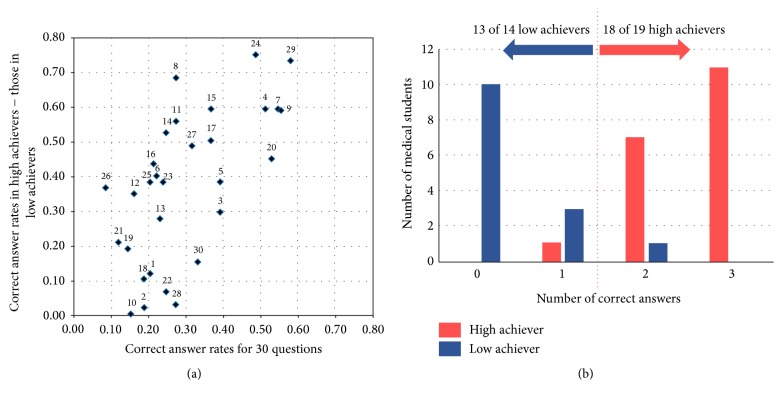
(a) Association between the learning effectiveness of KM education and the 30 multiple-choice questions (from number 1 to number 30). We adopted the proficiency index (PI), which is the difference between the high and low achievers in the correct answer rate for each question. The PI is calculated by subtracting the correct answer rate for the low achievers (*n* = 14) (vertical axis) from that in the high achievers (*n* = 19). Correct answer rates for each of the 30 questions are plotted on the horizontal axis. (b) Classification of high and low achievers according to correct answer rates for a combination of three questions (number 8, “five-element” theory, number 24, evidence-based KM, and number 29, two fields such as pattern-based diagnostic procedures [*four physical examinations of Kampo Medicine*] and pattern-based therapeutic theory [*ho-sho sotai*]). Note that 18 (94.7%) of the 19 high achievers correctly answered two or three of these three questions. On the contrary, 12 (92.3%) of the 13 low achievers gave only one or no correct answer to these three questions.

Regarding our investigation into which of the 30 multiple-choice questions most strongly affected the learning effectiveness of MSs, we calculated and then plotted the PI and correct answer rate for each of the 30 questions (sequentially from number 1 to number 30) on the vertical and horizontal axes, respectively, as shown in Figure 4(a), in order to identify the most robust combination of questions for classifying high and low achievers. We found that a combination of three questions (number 8, “five-element” theory, number 24, evidence-based KM, and number 29, two fields such as pattern-based diagnostic procedures [*four physical examinations of Kampo Medicine*] and pattern-based therapeutic theory [*ho-sho sotai*]) most accurately classified high and low achievers (Figure 4(b)). Namely, 18 (94.7%) of the 19 high achievers correctly answered two or three of these three questions. On the contrary, 12 (92.3%) of the 13 low achievers gave only one or no correct answer to these three questions.

## 4. Discussion

In this article, we described a web-based test system designed to provide an accurate assessment of the learning effectiveness of undergraduate education for KM among MSs. In addition to demonstrating success in the early validation phase, we identified the combination of test questions that could most accurately classify high and low achievers, which was an intriguing finding.

The World Federation for Medical Education advocates the integration of information and communication technology into the medical curriculum to enhance quality of care and enable continuous knowledge updating [11]. The use of the Internet as a teaching medium has increased rapidly in recent years. Among several tools available, the Moodle platform has been applied to various medical education systems, including virtual training and e-learning systems that can simulate actual rounds at emergency medical units and assist undergraduate physiology teachers and MSs alike [12, 13]. To the best of our knowledge, no studies have been conducted on an evaluation system for KM education using the Moodle platform. Therefore, we used the Moodle platform to develop and evaluate a web-based test measure of the learning effectiveness of KM education among MSs after KM lectures.

MSs ≥24 years of age and those who had been admitted to an undergraduate program scored higher on our web-based test compared with other MSs. For more than 5 years (data not shown), the same results have been found at our university using other measures, which supports the validity of our web-based test. Our results can be explained largely by the fact that MSs who had been admitted to an undergraduate program tended to be older (mean age 30.9 ± 2.4 years) than other MSs (mean age 22.9 ± 2.0 years) and in part by the fact that most of these MSs had some previous work experience or had studied humanities at another university. In terms of differences in social backgrounds, these types of MSs also tend to be more interested in Eastern culture, which plays a central role in KM learning [14] and thereby leads to higher proficiency levels.

Another advantage of the web-based evaluation system developed in this study is that it can compile portfolios regarding proficiency patterns for the 8-type fields of KM for individual MSs (Figure 1). Indeed, directly after the web-based test, we provided individual portfolios to all 117 MSs; this made it possible for both instructors and MSs to share information regarding both their strengths and weaknesses in terms of the 8-type fields of KM. Given the reported lack of consistency between undergraduate and graduate medical KM education at almost all of Japan's medical schools [5], our newly developed system is expected to serve as a robust tool that allows a better understanding of proficiency levels among MSs who will pursue graduate KM education elsewhere. Most Japanese physicians who did not take any KM classes during their undergraduate medical education learned about KM through self-study [15]. In this regard, our evaluation system could be particularly beneficial to MSs self-taught in KM.

One of the new findings that emerged from cluster analysis in this study was that high and low achievers had different proficiency patterns for the 8-type fields of KM. More importantly, many low achievers had particularly low scores for the history of KM, which suggests that historical context is extremely important for grasping a good understanding of KM. This seems reasonable, as KM theory has been constructed gradually over a long period of time [16]. Therefore, greater integration of education of the history of KM into current graduate and undergraduate education programs is needed. However, another possible explanation is that historical knowledge seems to be more easily forgotten than understanding of the unique theory of KM such as “*ho-sho sotai.*” This is also supported by the fact that someone of low achievers, students with low performance status on the web-based test, indeed attempted to learn the history of KM by rote. Further examinations are needed to clarify the relation between the proficiency levels for KM and the degree of concern about the history of KM.

It is known that the actual integration of hands-on practice for KM into the educational program was done in less than 20% of Japan's medical universities and colleges [6]. Thus, one limitation of the present study was that KM education was offered to MSs primarily in the form of classroom lectures. As well as hands-on practice for KM, integrating project- or team-based learning into the current education system is needed to allow MSs to better understand various aspects of KM [5, 17], one of which is holistic medicine wherein mind and body are regarded as unified. These effects on the proficiency levels would be evaluated objectively on the web-based system.

Another new finding that emerged from this study was that 31 (94%) of the 33 high and low achievers were correctly classified based on a combination of only three questions regarding “five-element” theory, evidence-based KM, and two fields such as pattern-based diagnostic procedures [*four physical examinations of Kampo Medicine*] and pattern-based therapeutic theory [*ho-sho sotai*]. This finding suggests that these components could be essential content in graduate and undergraduate education and may serve as robust predictors for the learning effectiveness of KM education; however, performance in terms of these three questions needs to be confirmed in independent cohorts from other medical schools. More interestingly, we found that some questions with a high PI belonged to various KM fields; therefore, instead of the KM fields, the actual question and answer choices may be important for understanding the proficiency levels of MSs during KM education.

In Japan, only a few medical universities and colleges have included KM-related questions on graduation and/or promotion testing [5], and no KM-related questions have been included on the National Medical Licensing Examination (NMLE) that MSs must pass to obtain a license to practice medicine in Japan. Whether KM-related questions could be set in NMLE or not may depend on the curriculum standardization for KM that is desired by many KM educators [6]. However, it is not easy to standardize the contents of education program for KM because of the presence of various schools. Rather, the close way to the curriculum standardization is to collect and accumulate a set of both questions regarding KM and the proficiency data of MSs who take them. In this regard, our accumulated data might serve as a useful source of information for the NMLE in terms of the inclusion of suitable questions regarding KM.

## 5. Conclusions

Our newly developed web-based evaluation system rapidly and objectively measured the learning effectiveness of KM education among individual MSs. Furthermore, cluster analysis using correct answer rates for questions on 8-type fields of KM correctly classified high and low achievers. The proficiency patterns of the high achievers differed from those of low achievers. In contrast, three major clusters were identified within the low achievers, all of whom had a low percentage of or no correct answers regarding the history of KM. These findings suggest that our newly developed web-based evaluation system can accurately evaluate the learning effectiveness of KM questions in MSs. This type of evaluation could improve the inconsistencies in Japan's KM education system. However, further studies are needed in order to gain deeper insight into the present findings.

## Figures and Tables

**Table 1:** Test scores.

	Points	
	Mean (standard deviation)	*P* values
Sex		NS
Male (*n* = 74)	29.3 (11.7)	
Female (*n* = 43)	31.7 (12.2)	
Age		*P* < 0.05^*∗*^
≥24 years old (*n* = 86)	28.8 (11.0)	
<24 years old (*n* = 31)	34.1 (13.6)	
Mode for entrance		*P* < 0.00001^*∗∗*^
Usual university entrance examinations (*n* = 67)	28.8 (11.2)	*P* < 0.00001^*∗∗∗*^
Admission based on recommendation (*n* = 40)	28.4 (10.4)	*P* < 0.00001^*∗∗∗*^
Admission to an undergraduate program (*n* = 10)	46.9 (9.2)	

^*∗*^Student's *t*-test.

^*∗∗*^ANOVA test.

^*∗∗∗*^Compared with admission to an undergraduate program by Scheffe test.

NS: not significant.
